# Alpha ERS-ERD Pattern during Divergent and Convergent Thinking Depends on Individual Differences on Metacontrol

**DOI:** 10.3390/jintelligence11040074

**Published:** 2023-04-19

**Authors:** Chunlei Liu, Yuhong Lin, Chaoqun Ye, Jiaqin Yang, Wenguang He

**Affiliations:** 1 School of Psychology, Qufu Normal University, Qufu 273165, China; 2 Key Laboratory of Modern Teaching Technology (Ministry of Education), Shaanxi Normal University, Xi’an 710062, China

**Keywords:** metacontrol, creative thinking, alpha ERS-ERD, divergent thinking, convergent thinking

## Abstract

The role of metacontrol in creativity is theoretically assumed, but experimental evidence is still lacking. In this study, we investigated how metacontrol affects creativity from the perspective of individual differences. Sixty participants completed the metacontrol task, which was used to divide participants into a high-metacontrol group (HMC) versus a low (LMC) group. Then, these participants performed the alternate uses task (AUT; divergent thinking) and the remote associates test (RAT; convergent thinking), while their EEG results were recorded continuously. Regarding their behavior, the HMC group showed superior creative performance in the AUT and RAT, compared with the LMC group. For the electrophysiology, the HMC group showed larger stimulus-locked P1 and P3 amplitudes than the LMC group. Furthermore, the HMC group exhibited smaller alpha desynchronization (ERD) than the LMC group at the initial stages of the AUT task, followed by a flexible switching between alpha synchronization and desynchronization (ERS-ERD) during the process of selective retention in the AUT. In addition, the HMC group evoked smaller alpha ERD during the initial retrieval and the backtracking process in the RAT, associated with cognitive control adaptability. The aforementioned results indicate that metacontrol reliably contributes to the idea generation process, and HMC individuals could flexibly adjust their cognitive control strategies according to the demand for creative idea generation.

## 1. Introduction

Creativity is a sought-after ability, which generates novel and appropriate ideas or products (Sternberg and Lubart 1996). Creative idea generation emphasizes the importance of flexibility of cognitive control, which can allow individuals to consider and recombine multiple unrelated conceptions along uncommon paths. This suggests that creative thinking may involve metacontrol (Zhang et al. 2020). Metacontrol could be defined as the ability to monitor and regulate cognitive control, which refers to adaptive, high-level control or regulation of cognitive control to optimize target-directed behavior (Eppinger et al. 2021; Hommel 2015; Kang and Chiu 2021). An increasing number of researchers have emphasized that creativity tends to involve a dynamical variation of cognitive control, which largely relies on the metacontrol ability (Bocanegra and Hommel 2014; Hommel 2015; Hommel and Wiers 2017). The purpose of the present study was to investigate how individual differences in metacontrol ability affected creative thinking.

### 1.1. Definition of Metacontrol

According to the metacontrol state model (MSM), the present metacontrol state, which engages in goal-directed behavior, varies between persistence and flexibility and modulates the extent of competition between alternatives and the degree to which the existing objective affects them (Hommel 2015, 2019; Zhang et al. 2020). Metacontrol persistence states could strengthen the top-down influence and mutual competition of target, as well as target-consistent, alternatives to make more targeted decisions, whereas metacontrol flexibility states could present with attenuating top-down guidance and mutual competition from alternative selection candidates (Hommel 2015; Hommel and Colzato 2017b; Mekern et al. 2019b). Metacontrol states are supposed to select corresponding control operations according to cognitive control requirements and the current goal. People with different metacontrol capabilities may greatly differ in keeping and readjusting metacontrol states under dynamic situations. High-metacontrol individuals may flexibly switch between persistent and flexible states to accommodate the current task, while low-metacontrol individuals cannot. For instance, high-metacontrol individuals exert more “persistent” automatic control with low cognitive control demands; in turn, they allow more “flexible” cognitive control under high cognitive demands (Zink et al. 2018). There has been evidence that a high-metacontrol group (i.e., high adaptive ability) can cope with varying levels of cognitive control demands with superior performance than a low-metacontrol group when it comes to low control requirements (Zink et al. 2018). Concerning the neural mechanism for adaptive control, response-locked P3 amplitude had striking differences between the high- and low-metacontrol groups, which reflected the response selection adaptation to changing cognitive control needs (Stock et al. 2016; Twomey et al. 2015; Verleger et al. 2005; Zink et al. 2018). There is no significant difference in either bottom-up perceptual and attentional selection processes (P1 and N1 ERPs), nor in conflict monitoring processes (N2 and N450 ERPs) (Zink et al. 2018). The study demonstrated that the neural mechanism of inter-individual differences in metacontrol ability was only related to the response selection capability reflected by the response-locked P3. This revealed that inter-individual differences in metacontrol ability are reflected in reduced top-down control under low cognitive control, which is needed to complete the task more efficiently. 

### 1.2. Association between Metacontrol and Creative Thinking

The ability to regulate metacontrol bias according to the intended goal and the current context seems to be critical to generate creative ideas. A persistence state might allow individuals to struggle between alternatives, and eventually, choose one option which was guided by the goal. On the contrary, a flexible state might lead individuals to try to flexibly switch between different tendencies and result in an equivocal decision (Colzato et al. 2022). Hence, more persistent metacontrol states would be thought to support a highly constrained search for the only correct answer and strongly inhibit other target-inconsistent answers, which would be shown to promote convergent thinking. However, more flexible states would allow one to search for many vaguely defined answers and rapidly activate the most creative idea to facilitate divergent thinking. Different metacontrol states (i.e., persistence or flexibility) would facilitate the development of different types of creative thinking (Goschke and Bolte 2014; Hommel and Wiers 2017; Zhang et al. 2020). Actually, creativity tasks cannot be regarded as pure measures of divergent thinking or convergent thinking. This is a complex process, with different levels of reliance on divergent or convergent thinking at different stages, requiring both flexibility and persistence. High-metacontrol individuals can adaptively allocate control resources in a way that is goal-directed and meets the needs of the situation, so they prefer flexibility or persistence to a varying extent and better complete creative tasks. In sum, moderating the bias of metacontrol along the persistence-flexibility dimension, based on the intended goal and current context, may be crucial for the generation of creative ideas (Mekern et al. 2019b).

The link between metacontrol and creativity can be indirectly supported by the following findings. Some studies have shown that positive emotions contribute to divergent thinking by stimulating a flexible state, and negative emotions facilitate convergent thinking through a persistent state (Baas et al. 2008; Chermahini and Hommel 2010, 2012; Dreisbach and Goschke 2004). Similarly, meditation impacted the generation of creative thinking by the metacontrol state (Colzato et al. 2012, 2015; Hommel and Colzato 2017a; Ma et al. 2021; Ullrich et al. 2021). In addition, divergent thinking and convergent thinking could be employed to induce flexible or persistent biases of information processing, respectively (Fischer and Hommel 2012; Ma and Hommel 2020). In brief, these findings demonstrate that metacontrol is considered a new perspective to make sense of the neural and functional mechanisms underlying creative thinking. Creative idea generation may be driven by the metacontrol mechanism, which adaptively adjusts cognitive control to a certain extent in situations with different control requirements (Zhang et al. 2020). The MSM, mediating stable persistence and flexible switching of cognitive control, has provided a theoretical model that can explain the impact of cognitive control on creative thinking, but there is still very little relevant empirical research and evidence of the link between metacontrol and creativity, and hardly any empirical evidence to understand their underlying neural mechanisms. 

### 1.3. Aims of the Study

Herein, the present study aimed to clarify the specific impact of metacontrol on creativity and the neurocognitive mechanism of metacontrol in creative idea generation. For this purpose, the participants were divided into high- and low-metacontrol groups through a modified version of the metacontrol task (Zink et al. 2018). The divergent and convergent thinking of both groups were tested by the alternate uses task (AUT, Fu et al. 2022) and the remote associates test (RAT, Shen et al. 2016), respectively. This procedure can examine whether metacontrol influences creativity by exerting control adaptively. In addition, the present study employed electroencephalography (EEG) to enable a direct examination of neural oscillations in a context where metacontrol affects creativity. EEG techniques allow us to examine the different cognitive sub-processes between the high- and low-metacontrol groups (Zink et al. 2018) and could also provide more accurate evidence for grouping. Overall, we examine creative performance differences between high- and low-metacontrol individuals. Two hypotheses were formulated: First, we expected that high-metacontrol individuals would allocate cognitive control resources on demand, therefore surpassing low-metacontrol individuals in low-control-demand tasks. Second, we anticipated that high-metacontrol individuals would be better at divergent thinking and convergent thinking performance. This could provide some direct proof of whether and how metacontrol influences creativity. 

## 2. Methods

### 2.1. Participants

A two-tailed power analysis was performed using G*Power 3.1.9.7 for Windows to determine the appropriate sample size required to detect statistical differences with an effect size of 0.5, a power of 80%, and a significance level of 0.05. The results showed that 34 participants were needed per group. Seventy undergraduate students were recruited to attend this experiment via online community postings and advertisements posted in Qufu Normal University (Qufu campus). Ten participants were not considered in further analyses: three due to task discontinuation, three due to poor data quality caused by too many eye-movement and head-movement artifacts, three due to equipment failures resulting in incomplete EEG recording, and one who did not understand the task correctly. The final sample included 60 participants (19 males), who were healthy, right-handed, and had a normal or corrected-to-normal vision. They were divided into an HMC (7 males, *M*_age_ = 20.14, *SD*_age_ = 2.01) and LMC group (12 males, *M*_age_ = 20.78, *SD*_age_ = 2.04). Written informed consent was obtained at the beginning of the experiment for each participant, and all participants were compensated CNY 35 in addition to a bonus obtained during the experiment. All study procedures were approved by the Research Ethics Board of Qufu Normal University.

### 2.2. Procedure

Participants were required to complete metacontrol tasks and creative tasks (the AUT and RAT) with EEG recording continuously. E-Prime 2.0 software was adopted (Psychology Software Tools, Inc., Sharpsburg, Pennsylvania, USA) to administer the task. Practice trials were carried out before each task to familiarize participants with the task rules and procedures. 

### 2.3. Task and Experimental Materials

#### 2.3.1. Metacontrol Task

According to the metacontrol task (Bocanegra and Hommel 2014), participants completed two distinct tasks regulated by the rules’ complexity: an easy task (i.e., low control demands) and a hard task (i.e., high control demands). Eight different stimuli were presented in both tasks randomly and equally, varying in shape (square or diamond), color (green or red), and size (small, approximately 2.5 cm diameter, or large, approximately 5 cm diameter).

**Figure 1:**
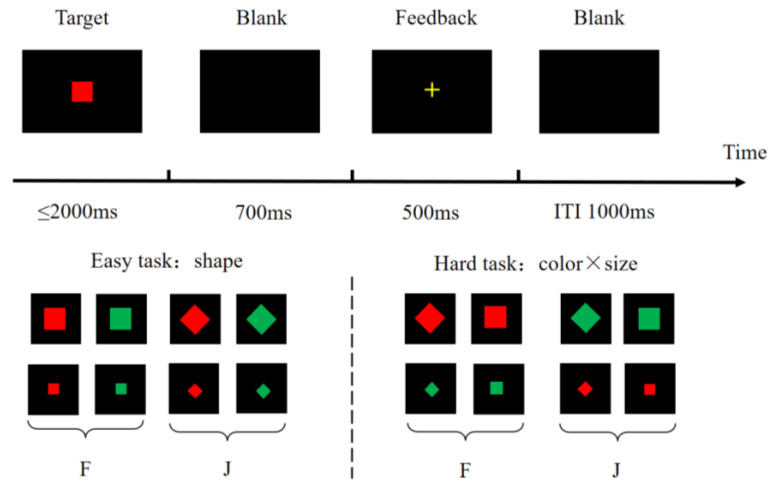
**Above**: Schematic time-course of the metacontrol paradigm. **Below**: Target stimuli for the easy and hard tasks.

Figure 1 shows the schematic time-course of the metacontrol task. In the hard task, participants were required to respond with both the size and color of the stimuli. They pressed the “F” key if the stimulus was big and red or small and green; otherwise, they pressed the “J” key. In the easy task, they were merely asked to distinguish the shape of the stimulus as either diamond or square, using the “F” or “J” key, respectively. Participants were expected to respond as rapidly and correctly as possible. They had to press the key in a maximum of 2000 ms; otherwise, an incorrect response was recorded. Subsequently, a black screen was displayed for 700 ms after response or time-out, and then feedback concerning their correctness appeared for 500 ms. After a 1000 ms black screen, the next trial began. The practice block consisted of eight trials to ensure understanding of the task. Following the practice block, participants completed five blocks (96 trials each) in both tasks. The hard task was performed first, followed by the easy task, to maintain the potential order effects constantly. 

To explore how metacontrol impacts creativity, we divided the individuals into the HMC and LMC groups according to a median split of the adaptability score. The HMC group is mainly characterized by high adaptability to easy and hard tasks, while the LMC group is characterized by low adaptability. The adaptability score was calculated by subtracting the performance ratio in the easy task and hard task, reflecting the performance differences between the two tasks. The performance ratio, which is the accuracy rate (ACC) divided by the hit reaction time (RT), reflects the performance of participants in metacontrol tasks. The larger performance ratio indicates relatively more accurate and/or rapid responses.

$$Adaptability\;Score={performance\;ratio}_{easy}-{performance\;ratio}_{hard}\;=\frac{{ACC}_{easy}}{{RT}_{easy}}-\frac{{ACC}_{hard}}{{RT}_{hard}}$$

#### 2.3.2. Creative Task

(1) The Alternative Uses Task (AUT)

**Figure 2:**
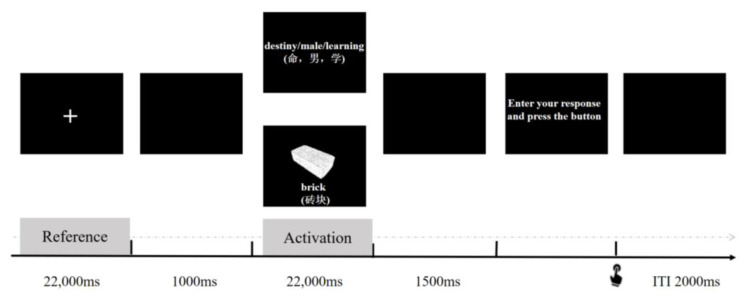
Trial structure of the alternative uses task (bottom) and the remote associates test (up).

The AUT serves as a test of divergent thinking (Guilford 1967). Participants need to create as many novel, creative, and practical ideas as possible for every object (e.g., a brick) within the stipulated time. As shown in Figure 2, the typical process can be described as follows: initially, each trial started with a fixation cross (22,000 ms; reference period), and then participants genereate as many ideas as possible on how to use the presented object (22,000 ms; activation period). Subsequently, participants were asked to choose the most original, unconventional use to enhance elaboration and evaluation during the process of creative thinking. At last, participants entered their ideas into an E-prime response box via keyboard. The following trial began after a 2000 ms interval (ITI). To assess their originality, we evaluated the participants’ answers based on the consensus assessment technique. Four trained raters used a five-point Likert scale ranging from 1 (“hardly original”) to 5 (“quite original”) to evaluate the originality of each idea. They had satisfactory inter-rater reliability (ICC (2, k) = 0.844). The final original score was calculated by averaging the ratings of the four raters.

(2) The Remote Associates Test (RAT)

For convergent thinking, we employed the Chinese RAT (Shen et al. 2016). Three clue words appeared on the screen (e.g., “命”, “男”, “学”, literally “destiny/male/learning”). Participants were asked to think of a target word (e.g., “生”, literally “person” in English), which can build a semantic bridge with the provided three clue words. This can be combined with the three clue words to form meaningful phrases (“生命”, “男生”, “学生”, literally “life/male/student”). To eliminate the additional impact of task difficulty, 15 RAT items of medium difficulty were selected. For the process, the fixation cross and each subsequent blank screen were presented for 22,000 ms and 1000 ms, respectively, followed by 22,000 ms RAT items. Participants were required to think of an answer and enter the answer into the response box. An interval of 2000 ms was included between items.

The order of the AUT and RAT tasks was counterbalanced among participants during this session.

### 2.4. EEG Recording and Pre-Processing

EEG data were recorded via a Brain Products GmbH 64-channel system configured to the 10–20 system. FPz and FCz were selected as the ground electrode and reference electrode, respectively. To avoid power line contamination, an online filter between 0.1 Hz and 100 Hz was applied to the EEG signals, as well as a 50 Hz notch filter. EEG signals were digitalized at 1000 Hz. All impedances remained below 5 kΩ.

EEG data were analyzed with Brain Vision Analyzer 2.1 software. The raw data were re-referenced to the average of the left and right mastoid electrodes and passed through the IIR filter from 0.1 Hz to 40 Hz and a notch filter at 50 Hz. Independent component analysis was performed to semi-automatically remove blinks and eye-movement artifacts from continuous EEG data (ICA; Infomax algorithm).

### 2.5. EEG/ERP Data Analysis

#### 2.5.1. ERP for the Metacontrol Task

Stimulus-locked and response-locked segments were separately formed with correct responses for all experimental conditions to analyze the metacontrol task. Stimulus-locked segments ranged from −500 ms to 1000 ms, and response-locked segments were 1000 ms (500 ms before and after the response, respectively). Before stimulus onset, a time window of 300 ms was used to perform the baseline correction for the stimulus-locked segments. For the response-locked segments, a baseline correction was performed ranging from 300 ms to 400 ms after the response. The segments with artifacts, due to eye blinks, eye movements, or muscle tension, were semi-automatically removed.

As ERPs, including the P1 and P3, are commonly assumed to best reflect the sub-processes of metacontrol (Stock et al. 2016; Zink et al. 2018, 2019), they were systematically analyzed. The P1 amplitudes were quantified in a time window of 75–135 ms over electrodes of P7 and P8, which were averaged across the electrodes used for subsequent analysis. The stimulus-locked P3 amplitudes were extracted at electrodes P7, P8, and Pz in a time window ranging from 320 to 470 ms, 280 to 440 ms, and 330 to 470 ms, respectively; these were averaged across these electrodes used for statistical analysis. The response-locked P3 amplitudes were measured at electrodes P7, P8, and Pz from −380 to −240 ms and were averaged and used for subsequent analysis.

#### 2.5.2. EEG Alpha TRP for AUT and RAT

For the AUT and RAT, after preprocessing the data and removing the artifacts, we further applied an FFT filter to the alpha frequency band (8–13 Hz) to filter EEG signals. We squared the filtered EEG signals to get power estimates and then (horizontally) averaged the band power values (µV^2^) for every single trial. We employed task-related power (TRP) changes to quantify brain activity during creative ideation (Benedek et al. 2011; Fink et al. 2006). To investigate whether the time-course of alpha TRP during idea generation could be affected by metacontrol, the pre-stimulus reference interval (i.e., 20,000 ms, from 1000 ms to 21,000 ms, presenting the fixation cross) and the activation interval (i.e., 20,000 ms, from 1000 ms after stimulus onset to 21,000 ms; see Figure 2) were split into ten isochronous time intervals of 2000 ms each (Rominger et al. 2018). 

We employed the following equation to quantify alpha TRP for each electrode i: TRP(i) = log (Powi, activation) − log (Powi, reference). The negative TRP values indicate the decreases in alpha power from the reference to the activation period (i.e., desynchronization), whereas positive TRP values reflect an increase (i.e., synchronization) (Pfurtscheller and Andrew 1999). Electrode positions were topographically aggregated to perform statistical analysis as follows: frontal left (F3, F1), frontocentral left (FC3, FC1), central left (C3, C1), centroparietal left (CP3, CP1), and parietal left (P3, P1) electrodes, and the corresponding electrodes in the right hemisphere.

### 2.6. Statistical Analysis

Given previous studies on metacontrol (Zink et al. 2018), we used a median split of an adaptability score to divide the participants into two groups. To verify the validity of grouping, we used repeated measures analysis of variance (ANOVA) to examine whether high- and low-metacontrol groups had different performances and cognitive sub-processes in the metacontrol task. Task (easy vs. hard task) was the within-subject variable, and Group (high- vs. low-metacontrol group) was the between-subject factor. The independent sample *t*-test method was used to analyze the creative task performance of AUT originality and RAT accuracy, respectively, to explore whether metacontrol affected creativity.

To examine the neurophysiological mechanisms underlying metacontrol-affected creative idea generation, repeated measures ANOVA was conducted for TRPs in the alpha (8–13 Hz) bands, Area (frontal, fronto-central, central, centro-parietal, parietal), Electrodes (two positions in each area of each hemisphere), Hemisphere (left vs. right), Stage (S1, S2, S3, S4, S5, S6, S7, S8, S9, S10) as the within-subject variables and Group (high- vs. low-metacontrol group) as a between-subject variable. Mauchly test of sphericity was used for all ANOVAs. The Greenhouse–Geisser correction was applied when the sphericity assumption was violated. 

## 3. Results

### 3.1. Metacontrol Task Results

#### 3.1.1. Behavioral Results

**Figure 3:**
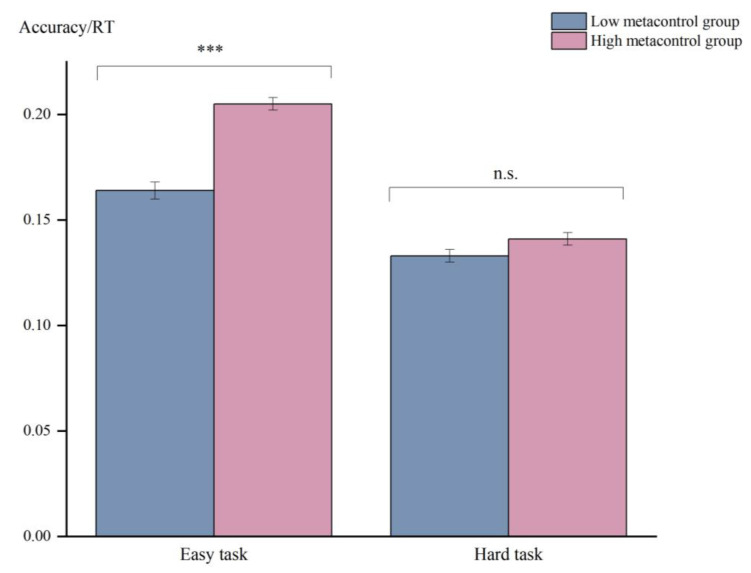
Performance ratio (accuracy divided by hit RT) of the low- and high-metacontrol groups for the easy and hard tasks. Error bars represent standard errors (*** *p* < 0.001, n.s., shows no significant difference).

Based on the results of the metacontrol tasks, Figure 3 exhibits the performance ratios of the HMC and LMC groups in the easy and hard tasks (i.e., accuracy divided by hit RT). The analysis showed a main effect of Task (*F*_(1,58)_ = 1183.00, *p* < 0.001, $\eta_{p}^{2}$ = 0.95), with significantly better performance in the easy task (*M* = 0.19, *SD* = 0.03) than in the hard task (*M* = 0.14, *SD* = 0.02). The effect of Group was also significant (*F*_(1,58)_ = 31.35, *p* < 0.001, $\eta_{p}^{2}$ = 0.35), with significantly better overall performance in the HMC group. The significant interaction effect of Task * Group indicated that the HMC group and LMC group had different performance under high and low control demand (*F*_(1,58)_ = 136.05, *p* < 0.001, $\eta_{p}^{2}$ = 0.70). The HMC group (*M* = 0.21, *SD* = 0.02) performed better than the LMC group (*M* = 0.16, *SD* = 0.02) in the easy task (*p* < 0.001), but this difference was not found in the hard task (*p* = 0.061). 

#### 3.1.2. ERP Results

**Figure 4:**
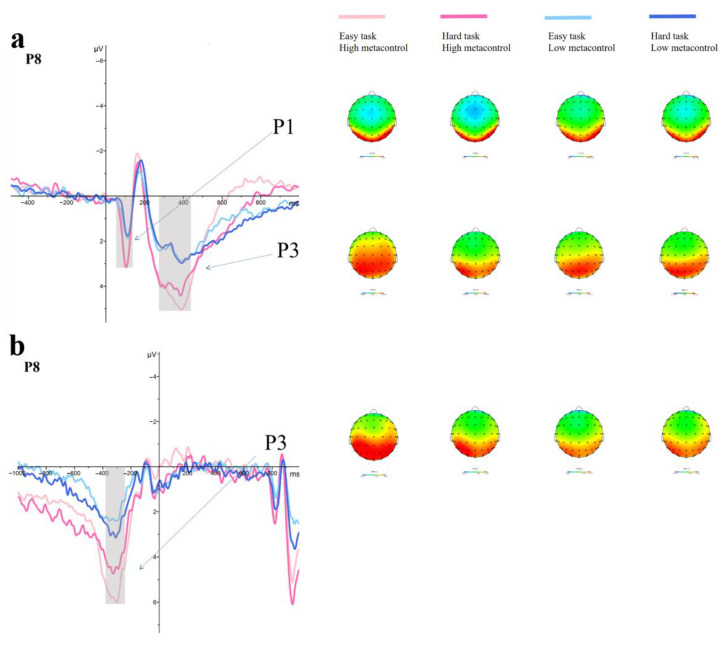
Stimulus-locked (**a**) and response-locked (**b**) grand-average waveforms from the P8 electrode and topographic maps for the metacontrol task.

Figure 4 shows the grand average wave and topographic plots of the metacontrol task. 

P1: There was a significant main effect of Group (*F*_(1,58)_ = 13.78, *p* < 0.001, $\eta_{p}^{2}$ = 0.19), with more positive P1 amplitude in the HMC group than in the LMC group. There was no main effect of Task (*F*_(1,58)_ = 0.21, *p* = 0.649), nor a Task * Group interaction (*F*_(1,58)_ = 0.51, *p* = 0.477).

Stimulus-locked P3: There was a significant main effect of Group (*F*_(1,58)_ = 5.40, *p* = 0.024, $\eta_{p}^{2}$ = 0.09) with more positive P3 amplitude in the HMC group than in the LMC group, in the absence of a main effect of Task (*F*_(1,58)_ = 0.04, *p* = 0.840). A significant Task * Group interaction (*F*_(1,58)_ = 5.26, *p* = 0.026, $\eta_{p}^{2}$ = 0.08) was observed, in which the amplitude of the P3 in the HMC group was significantly larger (*M* = 5.18, *SD* = 2.30) than that of the LMC group (*M* = 3.45, *SD* = 2.20) in the easy task (*p* = 0.004). However, there was no difference in the hard task (*p* = 0.174). 

Response-locked P3: There was a significant main effect of Group (*F*_(1,58)_ = 10.83, *p* = 0.002, $\eta_{p}^{2}$ = 0.16), with larger P3 amplitudes in the HMC group than the LMC group. There was no main effect of Task (*F*_(1,58)_ = 2.25, *p* = 0.139). Furthermore, a significant interaction effect Task * Group was found (*F*_(1,58)_ = 9.60, *p* = 0.003, $\eta_{p}^{2}$ = 0.14), showing that the HMC group had larger P3 amplitudes (*M* = 5.67, *SD* = 2.37) than the LMC group (*M* = 3.46, *SD* = 1.66) in the easy task, while there was no significant difference (*p* = 0.115) in the hard task.

### 3.2. AUT and RAT Results

#### 3.2.1. Behavioral Results

An independent sample *t*-test was used to examine whether there was a difference in creativity performance between the HMC and LMC groups. For AUT originality, it demonstrated a higher originality in the HMC group (*M* = 2.97, *SD* = 0.22) than the LMC group (*M* = 2.75, *SD* = 0.31; *t*_(58)_ = 3.19, *p* = 0.002, Cohen’s *d* = 0.84). For the RAT, it was also observed that the HMC group had higher accuracy (*M* = 61.15, *SD* = 13.13) than the LMC group (*M* = 50.24, *SD* = 12.89; *t*_(58)_ = 3.24, *p* = 0.002, Cohen’s *d* = 0.84). 

#### 3.2.2. EEG Alpha TRP Results

Time-course of alpha power during creative ideation in metacontrol individuals was performed to test the neurocognitive mechanisms of metacontrol affecting creativity. The ANOVA with between-subject factor Group (HMC group vs. LMC group), within-subject factor Stage (S1, S2, S3, S4, S5, S6, S7, S8, S9, S10), Hemisphere (left vs. right), Electrode (two positions in each hemisphere), and Area (frontal, fronto-central, central, centro-parietal, parietal) were carried out on alpha TRP values. 

**Figure 5:**
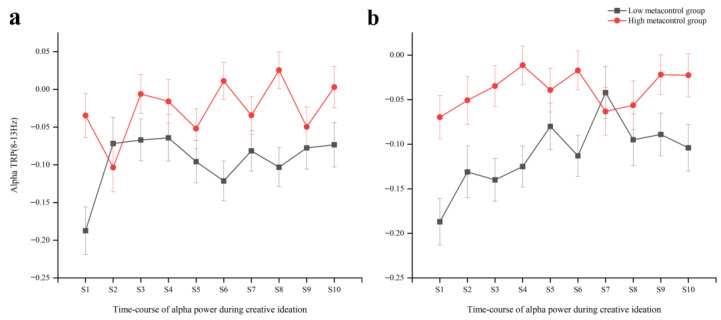
Means and standard error bars of task-related alpha power changes during the course of alternative uses task (**a**) and remote associates test (**b**).

For the AUT alpha TRP (Figure 5a), the ANOVA showed significant main effects of Area (*F*_(4,232)_ = 8.81, *p* = 0.001, $\eta_{p}^{2}$ = 0.13) and Stage (*F*_(9,522)_ = 2.20, *p* = 0.037, $\eta_{p}^{2}$ = 0.04), as well as a significant main effect of Group (*F*_(1,58)_ = 8.35, *p* = 0.005, $\eta_{p}^{2}$ = 0.13), with significantly larger alpha ERD in the LMC group than in the HMC group. The interaction Stage ∗ Group was also significant (*F*_(9,522)_ = 2.76, *p* = 0.010, $\eta_{p}^{2}$ = 0.05), which indicated that the HMC group and LMC group had different alpha ERD trends during the AUT. Bonferroni-corrected pairwise comparisons showed that there was a significant difference between the HMC group and LMC group in AUT-Stage1, AUT-Stage6, and AUT-Stage8. An ANOVA showed significant interactions of Stage ∗ Area (*F*_(36,2088)_ = 1.91, *p* = 0.034, $\eta_{p}^{2}$ = 0.03), Hemisphere * Electrode (*F*_(1,58)_ = 4.64, *p* = 0.035, $\eta_{p}^{2}$ = 0.07), and Area * Hemisphere * Electrode (*F*_(4,232)_ = 4.17, *p* = 0.010, $\eta_{p}^{2}$ = 0.07), and there were no other significant main effects or interactions observed (*p* > 0.05).

For the RAT alpha TRP (Figure 5b), the ANOVA showed a significant main effect for Stage (*F*_(9,522)_ = 2.53, *p* = 0.017, $\eta_{p}^{2}$ = 0.04), Area (*F*_(4,232)_ = 51.31, *p* < 0.001, $\eta_{p}^{2}$ = 0.47), and Electrode (*F*_(1,58)_ = 5.91, *p*= 0.018, $\eta_{p}^{2}$ = 0.09), as well as a significant main effect of Group (*F*_(1,58)_ = 9.75, *p* = 0.003, $\eta_{p}^{2}$ = 0.14), with significantly larger ERD in the LMC group than in the HMC group. The time-course of the HMC group alpha power showed significant larger ERD than the LMC group in the RAT-Stage 1 (*p* = 0.002), RAT-Stage 2 (*p* = 0.047), RAT-Stage 3 (*p* = 0.002), RAT-Stage 4 (*p* = 0.001), RAT-Stage 6 (*p* = 0.004), RAT-Stage 9 (*p* = 0.045), and RAT- Stage 10 (*p* = 0.025), as indicated by a significant interaction of Stage * Group (*F*_(9,522)_ = 2.24, *p*= 0.034, $\eta_{p}^{2}$ = 0.04). The interaction effects of Stage * Area (*F*_(36,2088)_ = 3.37, *p* < 0.001, $\eta_{p}^{2}$ = 0.06), Area * Electrode (*F*_(4,232)_ = 2.91, *p* = 0.037, $\eta_{p}^{2}$ = 0.05), and Stage * Hemisphere * Electrode (*F*_(9,522)_ = 2.57, *p* = 0.013, $\eta_{p}^{2}$ = 0.04) were also significant, while the other main effects and interactions were non-significant (*p* > 0.05).

In order to explore whether divergent and convergent thinking involve different neural and functional mechanisms, Task was used as a within-subjects factor in further analysis. Two significant interaction effects emerged: Task * Stage * Group (*F*_(9,522)_ = 1.95, *p* = 0.043, $\eta_{p}^{2}$ = 0.03), and Task * Stage * Area (*F*_(36,2088)_ = 2.64, *p* = 0.002, $\eta_{p}^{2}$ = 0.44). Specifically, the results revealed significant differences between the AUT and RAT tasks of LMC groups in stage 3 (*p* = 0.042), and significant differences between the AUT and RAT tasks of HMC groups in stage 8 (*p* = 0.040). The difference between the two tasks in stage 3 in the centro-parietal (*p* = 0.015) and the parietal (*p* = 0.020) and in stage 8 in the parietal (*p* = 0.013) was significant. The result revealed that a different characteristic trend of TRP regarding the time-course of creative ideation between AUT and RAT, which may reflect different neural and functional mechanisms. 

## 4. Discussion

The current study investigated the influence of metacontrol on creativity and its cognitive neural mechanism. Firstly, we checked whether the method of grouping was effective by examining the metacontrol task performance from behavioral and ERP data. The behavioral results showed that the HMC group was superior to the LMC group, which was reflected by higher accuracy and shorter response time, especially in the easy task. As already stated in previous studies, such as that of Zink et al. (2018), given the limited and exhaustible availability of control resources, the strategic adjustment of control against real control requirements optimizes the use of cognitive resources so that they work at maximum efficiency, which results in faster and less error-prone responses in the easy task. In addition, the ERPs show differences between the HMC and LMC groups. Specifically, the P1 amplitude of the HMC group was highly positive compared to the LMC group, which suggested the increase of attention allocation at early attention processing stages for the HMC group and reflected the differences in automatic processing patterns of physical information for stimuli between the HMC and LMC group (Herrmann and Knight 2001; Luck et al. 2000). Furthermore, the HMC group showed larger stimulus-locked P3 amplitudes than the LMC group, which suggested that the HMC group was superior during the monitoring and identification of stimuli. Similarly, the HMC group exhibited larger response-locked P3 amplitudes than the LMC group, which indicated that the HMC group was superior during the cognitive control and response selection (Kok 2001; Polich 2007). The fact that the HMC group evoked more positive stimulus-locked and response-locked P3 only in the easy task showed that the HMC group could exert less cognitive control with low control demands. The aforementioned finding is consistent with the previous reports that the P3 component reflects the adaptation to varying control demands during task processing (Stock et al. 2016; Twomey et al. 2015; Verleger et al. 2005, 2015; Zink et al. 2018). This result indicates that there are differences in the adaptability of cognitive control between HMC and LMC. HMC can reduce top-down control (P3 amplitude increases) for easy tasks and increase top-down control (P3 amplitude decreases) for hard tasks, while LMC cannot flexibly control their cognitive resources. Therefore, the behavioral and ERP results indicated that the HMC group is more efficient at allocating and utilizing control and performed better according to task demands, suggesting that the method of dividing the two distinct groups was effective.

Behavioral and electrophysiological tests were performed, and the corresponding results showed that metacontrol affects cognitive mechanisms during creative thinking. Specifically, the behavioral results revealed that the HMC group showed superior creative performance than the LMC group, with higher AUT originality scores and RAT accuracy. One explanation for this phenomenon was that the individuals with high metacontrol could find a better balance of flexibility and persistence by adapting their cognitive control to create more original solutions (Mekern et al. 2019a; Zhang et al. 2020). It was suggested that creative thinking depends upon the metacontrol states’ differences (Hommel 2015; Zhang et al. 2020); in other words, the optimal creative performance reasonably requires moderate flexibility (allowing individuals to consider diverse non-traditional ideas) or persistence (allowing individuals to dig deeper into the concept). The theory of event coding (TEC) by Hommel (2019) demonstrated that the overlapping features between stimulus and response might be weighted according to their goal-relevance to influence response selection. The particular metacontrol mode, varying between persistence and flexibility, could modulate to what degree multiple event files compete as well as the influence of current goals. Under a bias towards persistence, there are strong mutual competitions and strong top-down support, as required for convergent thinking, but both would be weak under a bias towards flexibility, as required for divergent thinking. HMC individuals could be more flexible to adjust their metacontrol state to suit the current context than LMC individuals, which might be more conducive to better completing creative tasks. Our findings suggested that the HMC group could manage the tradeoff between flexibility and persistence, which led to higher AUT originality and higher RAT accuracy. 

Since different strategies and cognitive processes are involved in creative idea generation (Finke et al. 1992; Schwab et al. 2014), we divided the ten stages of creative thinking into three processes according to the changing trend of alpha TRP values over time. Furthermore, we explored the time-course dynamic changes of alpha TRP values of the HMC and LMC groups during the process of idea generation (Agnoli et al. 2020; Rominger et al. 2019; Schwab et al. 2014). Specifically, stages 1–4 are defined as the initial retrieval process, stages 5–8 are the selective retention process, and stages 9–10 are the backtracking process. When they complete the AUT task, the differences in alpha power between the HMC and LMC groups in the first stage of creative ideation demonstrated significance, which indicates differences in the early processing and searching of visual stimuli (Benedek et al. 2014; Klimesch et al. 2007). The differences in P1 amplitude during the metacontrol task reflected the differences in the earlier attention processing and selective cognitive processing of physical stimuli between the HMC and LMC groups (Schneider et al. 2012). It may suggest why we observed the differences in information retrieval between the two groups during the first stage of creative ideation, possibly providing a cognitive mechanism underlying how metacontrol affects creative thinking. Moreover, the HMC group had flexible switching between alpha ERS and ERD in the process of selective retention, whereas the LMC group elicited an overall alpha desynchronization. This suggested that HMC individuals can repeatedly compare and select the generated ideas by inhibiting common ideas and producing more novel ideas, effectively evaluating and choosing the most creative one, which was crucial for high creative performance (Agnoli et al. 2020; Cheng et al. 2016). This process involves a dynamical variation of metacontrol states or adaptive cognitive control, which also fits with the claims of blind variation and selective retention (BVSR) (Campbell 1960; Simonton 2010), which means that the generation of creative thinking may involve the transformation of the blind variation (BV) process and the selective retention (SR) process. The BV process constantly generates new ideas, while the SR process makes individuals compare and choose generated ideas, and then retain appropriate views.

People with high metacontrol exhibited superior adaptable cognitive control during the process, which was also indicated by P3 in the metacontrol tasks (Stock et al. 2016). HMC individuals might pay selective attention to physical attributes of stimuli at the early stage, and then filter the information through attention. They need to continuously update working memory representations, recombine the information, and select and retain the most creative and appropriate answers by constantly breaking old connections and re-establishing novel ones. More flexible attention and more efficient memory storage and representation could allow individuals to select answers more efficiently. We inferred that adaptable cognitive control may be the mechanism for metacontrol affecting creativity, where the originality of generated ideas was further increased by their adaptive evaluation and elaboration at a different stage. 

Moreover, the differences in alpha power between the HMC and LMC groups at different stages of the RAT tasks might be relevant to the differences in their cognitive control adaptability. Specifically, the difference in the degree of alpha desynchronization was observed during the initial retrieval between the HMC and LMC groups, which may indicate the differences in cognitive control strategies during the period of earlier attention processing and memory retrieval. Meanwhile, differences in the degree of alpha desynchronization during the backtracking process were also detected between the two groups, which may be associated with idea elaboration and evaluation. The aforementioned results indicated that the differences in the initial information retrieval, as well as retrospective memory, may be the critical reason for the differences in RAT performance between HMC and LMC groups. This is also consistent with the differences in attentional processing and cognitive adaptability reflected in metacontrol tasks. This study’s results fitted with the notion that the selective processing of the target stimulus by the HMC individuals at the initial stage laid a foundation for subsequent problem-solving. The HMC individuals carried out adaptive control in the face of different cognitive needs, and adaptively adopted different cognitive strategies in different stages of creative thinking to better complete creative tasks.

To summarize, the HMC group had overall higher behavioral performance scores than the LMC group in metacontrol tasks, which is based on better performance in the easy task (i.e., low control demands). This is well in line with the concept of metacontrol (Eppinger et al. 2021; Hommel 2015; Kang and Chiu 2021). Individuals with better metacontrol abilities could monitor and regulate cognitive control and exert less control in the case of low control demands to optimize the use of the limited resource, thereby responding faster and more accurately (Bocanegra and Hommel 2014). We also found the behavioral group differences to be reflected by central measures of stimulus-response mapping processes (i.e., P3) (Polich 2007; Verleger et al. 2015). To explore the relation between metacontrol and creativity, we compared the creative performance difference between the HMC group and LMC group, and task-related EEG alpha power changes as a function of time. We found that the two groups activated different levels of alpha at different stages when facing AUT and RAT tasks. Moreover, the HMC group outperformed the LMC group in both AUT and RAT tasks. This allowed for the stronger and more powerful conclusion that individuals with better metacontrol abilities seemed to show adaptive control mechanisms in different stages during the time development of the thinking production to increase their response’s originality and accuracy.

There are also some limitations to this study, which could be addressed as follows. Firstly, a binary classification based on the median split was adopted in our study, while continuous data represented by metacontrol capabilities may be used in future research, and a model between metacontrol and creativity can be established. Moreover, further research could attempt to test whether our findings can be generalized to create tasks with more ecological validity and realistic demands. In addition, more attention can be paid to the causal contributions of metacontrol to creativity by manipulating different metacontrol states, concerning possible neurobiological mechanisms underlying the effects of the particular metacontrol bias between persistence and flexibility on creativity.

## 5. Conclusions

In conclusion, high metacontrol capability could promote creative thought via selective processing at the initial stage and carrying out adaptive control in face of different cognitive needs within different stages of creative thinking, which confirmed that divergent and convergent thinking involve different neural and functional mechanisms. Importantly, the link between metacontrol and creativity and their underlying neurocognitive mechanisms were elaborately elucidated. The present study claimed that creativity draws on an adaptive cognitive control process, and supported the metacontrol state model in the process of creativity.

## Data Availability

The data that support the findings of this study are available from the corresponding author, C.L., upon reasonable request.
